# Predicting postinfarct ventricular tachycardia by integrating cardiac MRI and advanced computational reentrant pathway analysis

**DOI:** 10.1016/j.hrthm.2024.04.077

**Published:** 2024-10

**Authors:** Pranav Bhagirath, Fernando O. Campos, Hassan A. Zaidi, Zhong Chen, Mark Elliott, Justin Gould, Michiel J.B. Kemme, Arthur A.M. Wilde, Marco J.W. Götte, Pieter G. Postema, Anton J. Prassl, Aurel Neic, Gernot Plank, Christopher A. Rinaldi, Martin J. Bishop

**Affiliations:** 1 School of Biomedical Engineering and Imaging Sciences, King’s College London, London, United Kingdom; 2 Department of Cardiology, Amsterdam University Medical Center, Amsterdam, The Netherlands; 3 Department of Cardiology, Royal Brompton & Harefield NHS Foundation Trust, London, United Kingdom; 4 Department of Cardiology, St. Thomas’ Hospital, London, United Kingdom; 5 Gottfried Schatz Research Center, Division of Biophysics, Medical University of Graz, Graz, Austria; 6 NumeriCor GmbH, Graz, Austria

**Keywords:** Ischemic cardiomyopathy, Late gadolinium enhancement, Ventricular tachycardia, Computational Modelling, ICD therapy

## Abstract

**Background:**

Implantable cardiac defibrillator (ICD) implantation can protect against sudden cardiac death after myocardial infarction. However, improved risk stratification for device requirement is still needed.

**Objective:**

The purpose of this study was to improve assessment of postinfarct ventricular electropathology and prediction of appropriate ICD therapy by combining late gadolinium enhancement (LGE) and advanced computational modeling.

**Methods:**

ADAS 3D LV (ADAS LV Medical, Barcelona, Spain) and custom-made software were used to generate 3-dimensional patient-specific ventricular models in a prospective cohort of patients with a myocardial infarction (N = 40) having undergone LGE imaging before ICD implantation. Corridor metrics and 3-dimensional surface features were computed from LGE images. The Virtual Induction and Treatment of Arrhythmias (VITA) framework was applied to patient-specific models to comprehensively probe the vulnerability of the scar substrate to sustaining reentrant circuits. Imaging and VITA metrics, related to the numbers of induced ventricular tachycardias and their corresponding round trip times (RTTs), were compared with ICD therapy during follow-up.

**Results:**

Patients with an event (n = 17) had a larger interface between healthy myocardium and scar and higher VITA metrics. Cox regression analysis demonstrated a significant independent association with an event: interface (hazard ratio [HR] 2.79; 95% confidence interval [CI] 1.44–5.44; *P* < .01), unique ventricular tachycardias (HR 1.67; 95% CI 1.04–2.68; *P* = .03), mean RTT (HR 2.14; 95% CI 1.11–4.12; *P* = .02), and maximum RTT (HR 2.13; 95% CI 1.19–3.81; *P* = .01).

**Conclusion:**

A detailed quantitative analysis of LGE-based scar maps, combined with advanced computational modeling, can accurately predict ICD therapy and could facilitate the early identification of high-risk patients in addition to left ventricular ejection fraction.

## Graphical abstract

**Figure:**
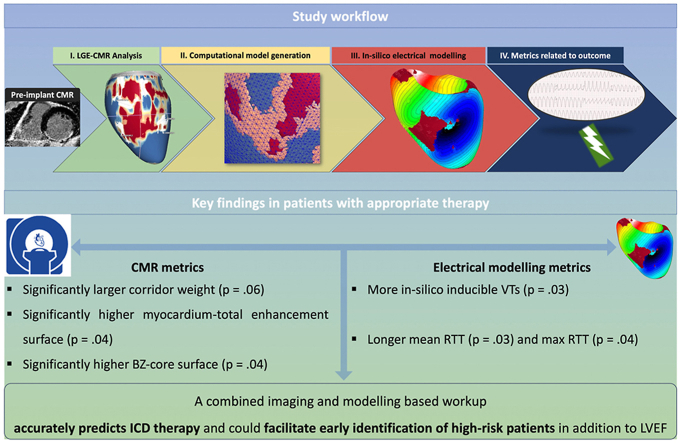

## Introduction

Identifying patients at risk of sudden cardiac death (SCD) after myocardial infarction remains a challenging task. Currently, reduced left ventricular ejection fraction (LVEF) is the most widely used marker to stratify patients for implantable cardiac defibrillator (ICD) implantation for the prevention of SCD.^1^ However, relatively few patients with an implanted ICD experience a life-threatening arrhythmic event.^2^^,^^3^ Early and accurate identification of high-risk patient groups, in whom an ICD would be effective, could improve quality of care.^4^ In addition, such a strategy stands to prevent unnecessary ICD implantation procedures, reducing procedure-related costs and complications.^5^ These findings warrant a more personalized characterization of the arrhythmic substrate to accurately identify patients at risk of SCD.

Recent developments in cardiac magnetic resonance (CMR) postprocessing have enabled a comprehensive and detailed characterization of late gadolinium enhancement (LGE) images. Commercially available tools can perform LGE analysis and be used to generate patient-specific enhancement (scar) maps. These maps can subsequently be used to calculate enhancement metrics such as size, location, and composition and thereby provide a noninvasive assessment of structural substrate complexity.^6^ However, the majority of such metrics are computed from 2-dimensional (2D) analysis of CMR slices. Given the intrinsic 3-dimensional (3D) nature of the electrical pathways through the infarcted regions, processing bespoke 3D quantitative metrics from LGE data may be required to fully characterize the proarrhythmic nature of the scar substrate.

In addition, LGE-derived maps can be used to directly generate anatomically patient-specific computational meshes and serve as an input for advanced personalized virtual arrhythmia induction protocols. Such approaches further augment the estimation of myocardial arrhythmogenic sensitivity by incorporating the functional effect (electrical instability) of the substrate,^7^ and these have been used to guide ventricular tachycardia (VT) ablation targets,^8^^,^^9^ predict ICD therapies in ischemic cardiomyopathy,^10^ and have been combined with machine learning approaches to stratify risk in cardiac sarcoidosis.^11^ Despite their success, the functional parameterization of the computational representation of electrophysiology models at the core of these methods is not patient specific and thus may not robustly extract all potentially proarrhythmic reentrant pathways that may be key for scar substrate complexity assessment and long-term risk stratification. Furthermore, the nature of the biophysically detailed electrophysiology models leads them to be highly computationally intensive, requiring dedicated off-site high-performance computing facilities. Before clinical translation, there is an urgent need to significantly reduce the computational time to be more commensurate with a clinical workflow.

In this study, we derive bespoke quantitative metrics describing the intricate topology of the scar substrate directly from 3D reconstructions of patient LGE data using the latest CMR analysis software for the prediction of ICD therapy in patients with ventricular electropathology following myocardial infarction. These 3D reconstructions are also used as input for our recently developed simulation-based approach to rapidly compute viable reentrant pathways through infarcted regions. The computationally lightweight nature of our approach allows us to quantitatively assess the proarrhythmic vulnerability of the substrate, which is related directly to patient outcomes in a highly computationally efficient manner.

## Methods

### Patient population

The study population consisted of a single-center prospective cohort of patients with an ischemic cardiomyopathy who had undergone CMR before ICD implantation. Consecutive patients undergoing primary and secondary prevention ICD implantation between May 2011 and January 2013 were prospectively enrolled. Ischemic cardiomyopathy was defined on the basis of standard criteria, including prior myocardial infarction, presence of any epicardial coronary artery stenosis ≥75%, or evidence of coronary revascularization with a scar pattern consistent with myocardial infarction on CMR imaging. *Primary prevention* was defined as ICD implantation to reduce SCD in at-risk individuals who had not yet experienced an aborted cardiac arrest or life-threatening arrhythmia. *Secondary prevention* was defined as ICD implantation in patients who already had experienced an aborted cardiac arrest or life-threatening arrhythmia. Study procedures were in accordance with the Declaration of Helsinki, and the study received approval from the local research ethics committee. All study subjects provided written informed consent.

### Image acquisition and postprocessing

The imaging workflow has been described previously.^12^ Briefly, 2D LGE-CMR images were acquired 10–15 minutes after contrast agent injection on a 1.5-T MRI scanner (Philips Healthcare, Best, The Netherlands). These images were semiautomatically segmented using ADAS 3D LV to create left ventricular (LV) endo- and epicardial contours (ADAS LV Medical, Barcelona, Spain).^13^ Color-coded pixel signal intensity maps were generated using a full-width half-maximum algorithm. The extent of variation in scar characteristics was evaluated in ADAS 3D LV by quantifying the border zone (BZ), scar core, and the number and weight of conduction corridors, that is, BZ surrounded by scar core. The resulting patient-specific enhancement model (exported as a series of shells) was postprocessed using an open-source meshing program (Meshtool)^14^ and custom-written python scripts to create volumetric meshes. Custom-written scripts were used to calculate metrics describing the 3D topology scar substrate, specifically the interface areas between healthy myocardium and total enhancement (BZ + core) and between BZ and core. Subsequently, fiber orientations were assigned within the myocardium by using a previously described rule-based approach.^15^ An overview of the studied measures can be found in the Online Supplement.

### Simulation workflow

In silico VT simulations were performed using a recently developed state-of-the-art tool Virtual Induction and Treatment of Arrhythmias (VITA).^16^ The first clinical validation of this tool was performed in a retrospective cohort of patients with VT ablation and enabled a noninvasive and robust assessment of VT substrate complexity.^17^ Briefly, a fast eikonal model was used to sequentially pace the ventricle at 17 sites (1 per American Heart Association segment). Splitting of the paced wavefront was used to automatically identify electrically isolated isthmuses through the scar. The corresponding reentrant pathways involving the identified isthmus were computed by simulating unidirectional block from the isthmus exit/entrance site. This is followed by computation of the round trip time (RTT), the computational equivalent of the cycle length, for each identified circuit. A cutoff of 50 ms was defined, where only circuits above this threshold were taken forward for further analysis. This RTT value could be considered to be too low, as VTs on average have a maximum cycle length of ∼200 ms. However, the aim of this investigation was to identify all potentially arrhythmic foci that could use small circuits and lead to polymorphic VT or ventricular fibrillation. Therefore, a small RTT enables the identification of all possible arrhythmogenic channels. Finally, duplicate VTs induced from different pacing sites were filtered out by calculating correlation coefficients on cyclically aligned activation time maps. As VITA is an algorithm based on scar topology, it represents a robust method of automatically extracting all anatomical isthmuses and subsequent viable reentrant pathways through the scar substrate, which may be missed with conventional virtual simulation approaches. Furthermore, the utility of VITA, being based on an eikonal method of simulating electrical propagation, allows for a significant reduction in compute time compared to biophysically detailed approaches.

Application of VITA to each computational model resulted in 4 simulation metrics derived from each simulation (for all 17 pacing site locations): total number of VTs induced from all pacing sites, number of unique VTs induced, and mean and maximum RTTs of all unique VTs. A more detailed description of the 4 studied measures is provided in the Online Supplement.

### Clinical follow-up

All patients underwent either ICD implantation or ICD implantation combined with cardiac resynchronization therapy device implantation. All devices were capable of storing electrograms that met the criteria for detection. A standardized ventricular arrhythmia detection and therapy algorithm was used: ventricular arrhythmias with heart rate >170 beats/min were detected (detection count 16 intervals) and treated with antitachycardia pacing (ATP). In case of ATP failure, the device was configured to deliver shock therapy. Ventricular arrhythmias with heart rate >210 beats/min were detected (detection count 24 of 30 intervals) and treated with shock therapy. Supraventricular tachycardia detection discriminators were enabled according to the recommendations of device manufacturers (St. Jude Medical, Inc., St. Paul, MN, and Medtronic, Minneapolis, MN; onset: 81% for Medtronic devices and 18% for St. Jude Medical devices; stability: 40 ms for both types of devices; morphology: passive). Patients were followed up at 3-month intervals. At each follow-up visit, device interrogation was performed, and all recorded events were reviewed by an experienced device physiologist and a trained electrophysiologist, blinded to the CMR data.

### Statistical analysis

Statistical analysis was performed using IBM SPSS Statistics (version 26) and Python v3.7.4 (Python Software Foundation, Wilmington, DE). Continuous variables were expressed as mean ± SD. The first episode of appropriate ICD therapy or sustained VT was considered the primary end point and used for quantifying all associations. Comparisons between the groups were made using the Fisher exact test for categorical variables and the Mann-Whitney *U* test for continuous variables. A *P* value of <.05 was considered to be statistically significant. Cox proportional hazards regression models were used to test the univariable association between the prespecified variables of interest and the primary end point. Multivariable models were used to determine the independent predictors of the primary end point (those significantly associated with the outcome of interest in univariable analyses; *P* < .05) including standard clinical metrics used to currently assess requirement for an ICD (LVEF and LV end-diastolic volume). Associations were reported as hazard ratios (HRs) and their corresponding 95% confidence intervals (CIs). Receiver operating characteristic curves were plotted to identify variable cutoff points with 90% sensitivity to discriminate the primary end point. The estimated cutoff values were used to reclassify and dichotomize the study subjects into high- and low-risk categories for the chosen imaging and simulation metrics to predict the primary end point. Subsequently, Kaplan-Meier survival curves were plotted to study the cumulative event rates between the groups of participants.

## Results

Forty patients (34 male 85%) were included for analysis with a mean follow-up of 7.34 ± 3.6 years. Of these, 26 patients (65%) had a device for primary prevention. All patients were on optimal heart failure and antiarrhythmic therapy before device implantation. In the interim period, patients had no further infarcts, coronary revascularizations, or catheter ablation procedures. Seventeen patients met the primary end point during the follow-up period, requiring device therapy in the form of ATP, biphasic shock, or a combination. The mean LVEF was 28.9% ± 11.6% without any significant differences between the 2 patient groups. Baseline characteristics are listed in Table 1.

**Table 1:** Baseline characteristics

Characteristic	Entire cohort (N = 40)	Therapy (n = 17)	No therapy (n = 23)	*P*
Age (y)	73.3 ± 8.3	73.8 ± 6.4	73 ± 9.6	.60
Men	34 (85)	15 (88)	19 (83)	
LV ejection fraction (%)	29 ± 11.4	26.2 ± 11.7	30.9 ± 11	.31
LV EDV (mL)	234 ± 67	250 ± 66	222 ± 65	.24
LV ESV (mL)	171 ± 69	188 ± 72	158 ± 64	.27
LV mass (g)	114 ± 24	117 ± 22	111 ± 25	.29
Prevention	Primary: 26 (65)	Primary: 9 (53)	Primary: 17 (74)	
	Secondary: 14 (35)	Secondary: 8 (47)	Secondary: 6 (26)	
Therapy	17 (42.5)			

Values are presented as mean ± SD or n (%).

EDV = end-diastolic volume; ESV = end-systolic volume; LV = left ventricular.

### Imaging and simulation metrics

Of the total 7 imaging metrics quantified, only 2 demonstrated significant differences between the event and nonevent groups (Table 2). Specifically, the interface areas between BZ and core (76 ± 26.7 vs 55.2 ± 27; *P* = .04) and between healthy myocardium and total enhancement (103.8 ± 34 vs 77.4 ± 33; *P* = .04) were significantly higher in patients with an event than in those without an event. Total corridors were comparable between both groups (6.53 ± 7.9 vs 4.6 ± 4; *P* = .38). Corridor weight demonstrated a trend toward higher mass in the event group (2.7 ± 2.1 g vs 1.6 ± 1.4 g; *P* = .06).

**Table 2:** Comparison of imaging and simulation metrics between therapy and no therapy groups (N = 40)

Variable	Event (n = 17)	Event-free (n = 23)	*P*
Imaging			
Border zone (g)	12.46 ± 6.2	9.46 ± 3.6	.14
Core (g)	8.09 ± 4.8	5.89 ± 4.0	.08
Total corridors	6.53 ± 7.9	4.57 ± 4.0	.38
Corridor weight (g)	2.74 ± 2.1	1.57 ± 1.4	.06
Total enhancement (g)	20.55 ± 10.3	15.35 ± 6.5	.08
Interface between healthy myocardium and scar (cm^2^)	103.76 ± 34	77.44 ± 33	.04∗
Interface between border zone and core (cm^2^)	75.96 ± 26.7	55.17 ± 27	.04∗
Simulation			
Total VTs	51.59 ± 46.1	29.74 ± 29.1	.08
Unique VTs	6.65 ± 4.1	4.13 ± 3.3	.03∗
Mean RTT (ms)	116.24 ± 49.4	78.65 ± 38.5	.03∗
Maximum RTT (ms)	194.35 ± 102	111.35 ± 74.5	.01∗

Values are presented as mean ± SD.

RTT = round trip time; VT = ventricular tachycardia.

∗ Significant difference (*P* < .05).

**Figure 1:**
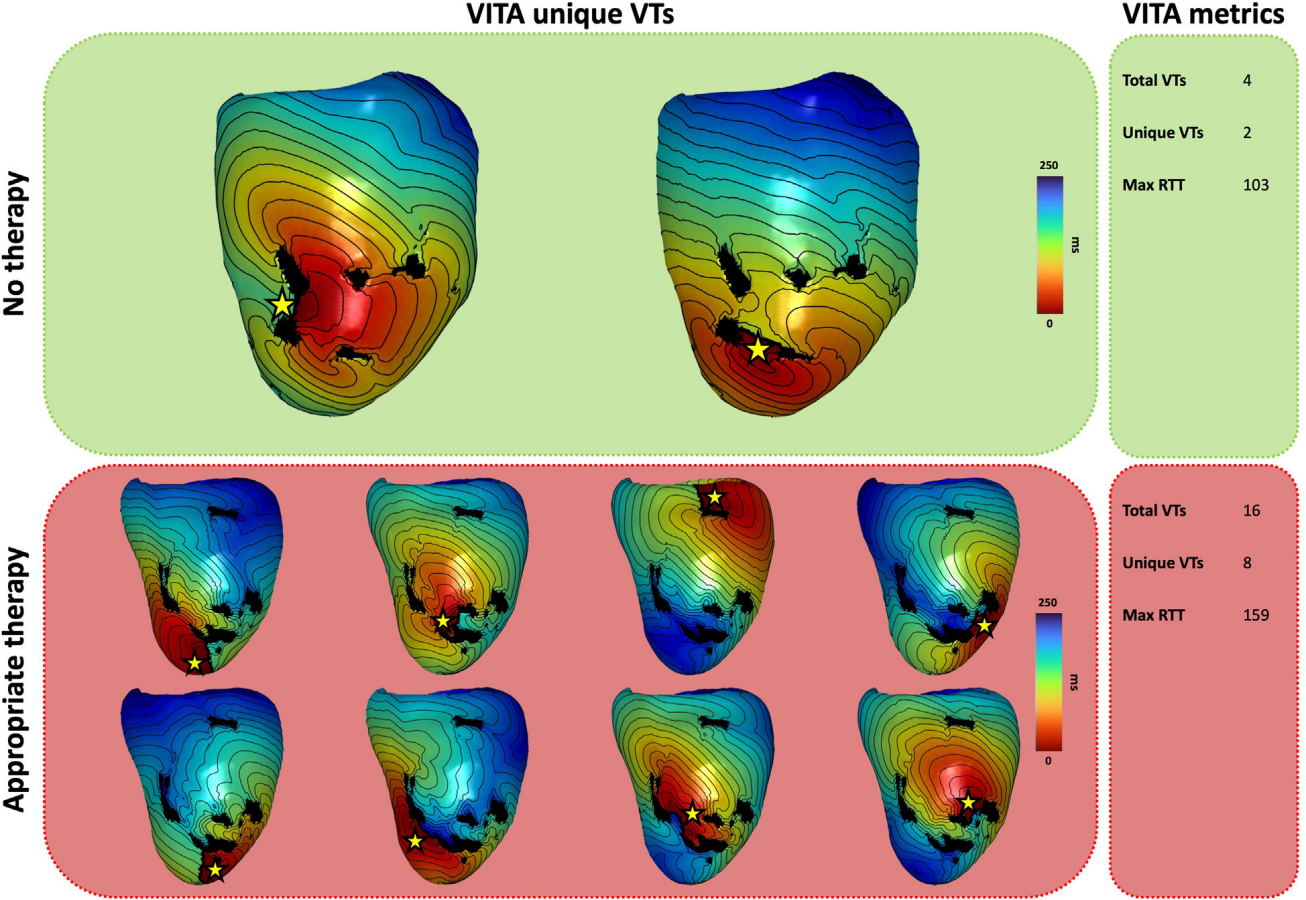
Differences in computational modeling metrics between patients with and without appropriate device therapy. Virtual Induction and Treatment of Arrhythmias (VITA) metrics–based differences between patient without (**top panel**) and with (**bottom panel**) appropriate device therapy. A significantly larger number of VITA-derived ventricular tachycardias (VTs) can be observed in patients who received appropriate therapy. The *yellow star* indicates the area of VT initiation, and the *black area* depicts scar core. In addition, a significantly larger round trip time (RTT) was observed in patients receiving appropriate device therapy.

In terms of simulation metrics, patients with an event had more unique VTs (6.7 ± 4.1 vs 4.1 ± 3.3; *P* = .03) during follow-up than did those without an event. Both mean RTT (116.2 ± 49.4 ms vs 78.7 ± 38.5 ms; *P* = .03) and maximum RTT (194.4 ± 102 ms vs 111.4 ± 74.5 ms; *P* = .01) were significantly higher in patients with an event (Figure 1).

### Predictors of device therapy

Univariable Cox regression (Table 3) revealed that multiple imaging and simulation metrics were significantly associated with an event. BZ mass was significantly associated with an event in univariable analysis (HR 1.51; 95% CI 1.02–2.22). Comparable associations were found for the 2 interface metrics: healthy myocardium with scar interface area (HR 2.12; 95% CI 1.29–3.49) and BZ with core interface area (HR 1.78; 95% CI 1.1–2.88). Only the healthy myocardium with scar interface metric remained a significant predictor in multivariable analysis whereas the remaining metrics, for example, scar core, BZ, corridors, and corridor weight, did not show a significant association for predicting an event (Table 3).

**Table 3:** Univariable and multivariable regression analysis of appropriate ICD therapy

Variable	Hazard ratio	95% confidence interval	*P*
Univariable			
Border zone	1.51	1.02–2.22	.04∗
Core (g)	1.33	0.88–1.99	.18
Total corridors	1.4	0.97–2.02	.07
Corridor weight	1.44	0.93–2.22	.10
Total enhancement	1.47	1.0–2.16	.05
Interface healthy-scar (per 10 cm^2^)	2.12	1.29–3.49	<.01∗
Interface border zone-core (per 10 cm^2^)	1.78	1.1–2.88	.02∗
Total VTs	1.53	1.05–2.23	.03∗
Unique VTs	1.56	1.02–2.37	.04∗
Mean RTT	1.59	1.04–2.43	.03∗
Maximum RTT	1.61	1.03–2.5	.04∗
Multivariable			
Border zone	1.82	0.8–4.11	.15
Interface healthy-scar	2.79	1.44–5.44	<.01∗
Interface border zone-core	1.53	0.86–2.7	.15
Total VTs	1.34	0.89–2.03	.16
Unique VTs	1.67	1.04–2.68	.03∗
Mean RTT	2.14	1.11–4.12	.02∗
Maximum RTT	2.13	1.19–3.81	.01∗

RTT = round trip time; VT = ventricular tachycardia.

∗ Significant difference (*P* < .05).

**Figure 2:**
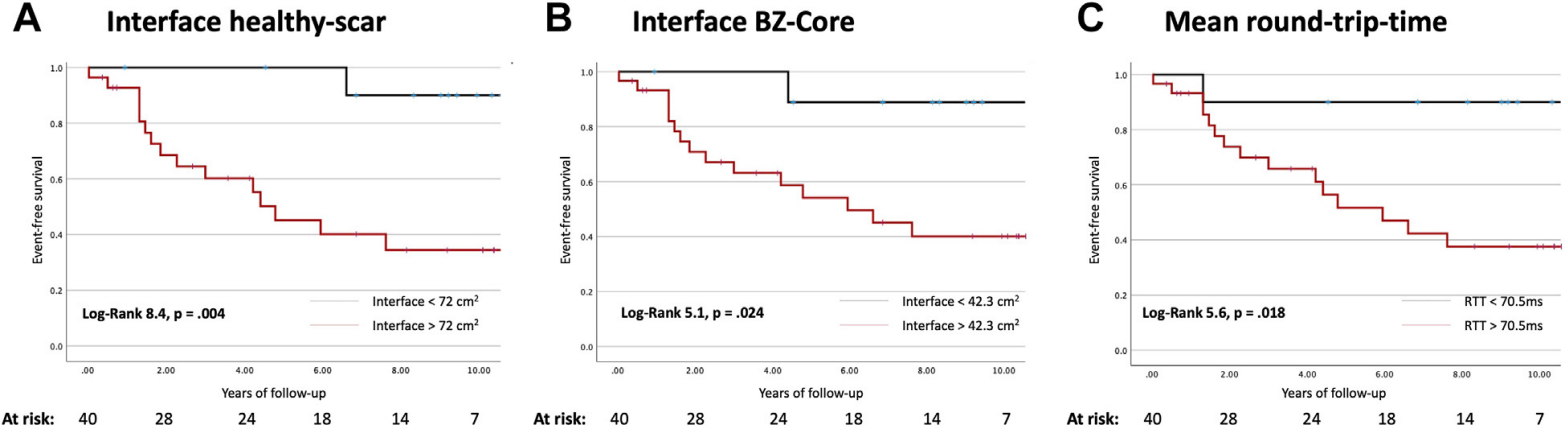
Kaplan-Meier curves for appropriate device therapy. Patients with a round trip time of >70.5 ms (**A**), an interface area between healthy myocardium and scar of >72 cm^2^ (**B**), and an interface area between border zone (BZ) and core of >42.3 cm^2^ (**C**) had a significantly higher number of ventricular arrhythmia events requiring device therapy.

All VITA metrics demonstrated a significant association with an event in univariable analysis: total VTs (HR 1.53; 95% CI 1.05–2.23; *P* = .03), unique VTs (HR 1.56; 95% CI 1.02–2.37; *P* = .04), mean RTT (HR 1.59; 95% CI 1.04–2.43; *P* = .03), and maximum RTT (HR 1.61; 95% CI 1.03–2.5; *P* = .04). All associations remained significant in multivariable analysis (Table 3), with the exception of total VTs. Kaplan-Meier analysis (Figure 2) showed a significantly higher event rate in patients with an interface area between healthy myocardium and scar of >72 cm^2^, in those with an interface area between BZ and core of >42.3 cm^2^, or in those with a mean RTT of >70.5 ms.

### Primary vs secondary prevention

In the primary prevention group, 34% of patients (n = 9 of 26) received appropriate device therapy compared with 57% of patients in the secondary prevention group (n = 8 of 14). The imaging metrics were comparable between the 2 patient groups and did not show any discriminatory power for appropriate therapy (Online Supplemental Table S1). Patients with an event in the primary prevention group demonstrated a nonsignificant trend toward a larger interface between healthy myocardium and scar (83.14 ± 32.4 vs 73.07 ± 24.5; *P* = .07) and between BZ-core (58.88 ± 25.4 vs 52.16 ± 21.8; *P* = .09) as compared with patients without an event.

In the secondary prevention group, all 4 VITA metrics were comparable between patients with and without an event. However, in the primary prevention group, patients with an event had a significantly higher number of total VTs (39.31 ± 42.8 vs 24.82 ± 24.4; *P* = .01), unique VTs (5.23 ± 4.5 vs 3.71 ± 3.5; *P* = .02), and maximum RTT (124.96 ± 72 vs. 98.29 ± 61.2; *P* = .02) than did patients without an event. Cox regression analysis demonstrated no significant associations of these parameters with an event.

## Discussion

This is the first study to report the utility of a *quantitative* workflow in predicting device therapy by using imaging- and simulation-derived novel metrics of ventricular substrate complexity. The results highlight that patients with appropriate device therapy had larger surface interface areas of enhancement regions with surrounding healthy myocardium, as quantified by 3D reconstructions of the scar substrate from LGE imaging. Furthermore, these patients had more in silico inducible VTs with longer RTTs indicating larger and more vulnerable circuits, as extracted with our novel simulation-based path finding algorithm applied directly to the 3D reconstructions. Finally, Cox regression analysis demonstrated the independent association between substrate interface areas and simulation metrics toward predicting appropriate device therapy.

### Imaging-based SCD and device therapy prediction

Multiple imaging studies have attempted to identify imaging biomarkers predictive of postinfarct malignant arrhythmias.^18^, ^19^, ^20^, ^21^, ^22^ There is increasing evidence that volumetric parameters, such as LVEF^20^ and LV end-diastolic volume,^21^ are unable to robustly identify high-risk patients. In recent years, multiple studies have evaluated LGE enhancement characteristics, such as scar core^23^ and BZ masses,^2^^,^^6^^,^^24^ to predict ventricular arrhythmias and suggested analysis methods and cutoff values to identify patients at risk of SCD.^25^^,^^26^ In addition, advances in postprocessing algorithms have seen the emergence of conduction corridors or channels in predicting postinfarct ventricular arrhythmia^27^ and guiding VT ablation.^28^, ^29^, ^30^ In line with these results, the present study demonstrates that conventional imaging parameters such as ejection fraction and ventricular mass and volumes lack the ability to distinguish between patients at higher risk of device therapy. In addition, BZ, while demonstrating significant univariable association, was not shown to be independently associated with the end point in multivariable analysis. Corridor weight also showed an important trend between event and nonevent groups, but did not achieve significance for prediction in time-to-event analysis.

### Predictive value of 3D metrics

Despite the rather weak association of macroscopic LGE-derived metrics, interestingly our analysis uncovered that other, more intricate, 3D metrics demonstrated a stronger association with the end point. Such metrics provide enhanced characterization of the complex topology of the infarct in 3D space. Postinfarct ventricular arrhythmia substrate is often a complex structure, traversing through the myocardium with multiple entrances and exits^31^ and its complexity cannot truly be appreciated using conventional 2D parameters. Intricate imaging metrics, such as the interface area between myocardium and scar as well as between BZ and core, may provide a more useful measure of the proarrhythmic functional implications of the structural substrate.

Clinical application of this metric has demonstrated its value in predicting arrhythmic events.^13^^,^^32^ Initially performed using a 2D slice reconstruction method in nonischemic cardiomyopathy, recent investigations have shown the utility of this metric, for the first time quantified fully as a 3D surface after 3D model reconstruction, in predicting arrhythmia recurrence after postinfarct VT ablation. In line with these results, the present study demonstrates the added value of these newer 3D interface metrics in successfully identifying patients at a higher risk of ICD therapy. In addition to serving as a robust predictor of arrhythmic events, interface can be applied in conjunction with computational modeling to provide novel functional and mechanistic insights into arrhythmogenic mechanisms.^32^ It can be hypothesized that the scar-BZ interface provides an estimate of the reentrant circuit length while the myocardium-BZ interface reflects the surfaces most likely to initiate unidirectional block. These results emphasize the importance of visualizing and thereby characterizing the substrate as a 3D entity instead of the currently applied 2D approach.

### Electrophysiological modeling to augment image-based prediction

Computational modeling can augment the insights from imaging-derived metrics by providing the functional implications of structural anatomy of the substrate. The numerous applications of monodomain computational modeling have provided novel insights into ventricular electropathology. These models have demonstrated promising results in identifying VT ablation targets,^8^^,^^9^ predicting SCD risk in hypertrophic cardiomyopathy,^33^ and improving assessment of ventricular arrhythmias in patients with cardiac sarcoidosis.^11^ Furthermore, a monodomain simulation–based computational study has shown that simulation metrics are more sensitive in predicting appropriate device therapy than conventional clinical parameters.^10^ In this study, we used our simulation approach to extract out a series of quantitative metrics that sought to characterize scar complexity, potentially providing more useful information compared to previous binary arrythmia-positive/arrythmia-negative simulation outputs.^10^ The in silico simulation findings in this study revealed that patients receiving device therapy had an extensive, more complex substrate as objectified by a larger number of in silico inducible VTs and longer mean and maximum RTTs. These results extend the evidence base for computational modeling and reinforce its reliability. This growing body of evidence proves the value of an integrated imaging and modeling strategy toward personalized arrhythmia risk assessment. Future (prospective) studies should investigate the clinical benefit of such a personalized selection and treatment approach.

### Advantages and future perspectives of VITA

VITA computes VT circuits using the RTT, the smallest (clinically) viable circuit. Although biological rhythms would have cycle lengths upward of 160 ms, circuits identified within the range of 50–160 ms could potentially become arrhythmogenic, for example, with the progression of underlying cardiomyopathy or exacerbate an existing arrhythmic focus, such as premature ventricular contractions or nonsustained VT, deteriorating into ventricular fibrillation. Therefore, in addition to providing clinically viable circuits, VITA provides additional “functional” complexity of the substrate by identifying circuits with RTT < 160 ms.

All previous simulation studies that have attempted to use virtual arrhythmia induction protocols on image-based anatomically patient-specific models to stratify risk have used monodomain approaches to represent tissue electrophysiology. These computationally intensive simulations require high-resolution computational models and take 1–2 days on a dedicated off-site high-performance computing facility to perform a full in silico VT induction protocol.^16^ Our VITA approach is built on a reaction-eikonal infrastructure, thereby benefitting from computationally lightweight models and highly rapid simulation times, meaning a comprehensive VT circuit assessment can be performed in ∼30 minutes on a standard desktop. Ultimate clinical translation of these computational methodologies requires further validation and testing on larger cohorts, containing many hundreds of patients, where multiple simulations and parameter sweeps for uncertainty analysis are necessary to robustly demonstrate their utility before uptake. The approach presented here not only provides a valuable tool for obtaining metrics that are strongly associated with events but is also commensurate with the use in a clinical workflow and/or in larger cohort studies. In addition, VITA could be further personalized by incorporating electrocardiogram data from individual patients. The integration of electrocardiogram–derived sinus rhythm characteristics into VITA could provide deeper insights into myocardial substrate characteristics and arrhythmogenicity on an individualized level and will be explored further in subsequent research using VITA-based computations.

### Limitations

The main limitation of this study is the relatively small patient cohort and its influence on the statistical robustness, most notable when subdividing the group in primary and secondary prevention cohorts. However, considering the extremely long follow-up and high number of events, the statistical analysis could be performed with sufficient accuracy. In addition, this is a comparatively large cohort performing personalized computational modeling in a patient cohort with implantable devices. The novel insights gained from this study about ventricular electropathology will be useful in informing future trials. Finally, this study investigated the utility of imaging and simulations to predict device therapy in ICD carriers. However, a far more profound clinical problem is the occurrence of SCD in patients currently not qualifying for devices. Future (prospective) application of VITA will focus on patients who do not qualify for a device on the basis of the current guidelines.

## Conclusion

LGE-based scar maps, combined with advanced computational modeling, enable a comprehensive, noninvasive assessment of ventricular electropathology. A combined workup can accurately predict ICD therapy and could thereby facilitate the early identification of high-risk patients in addition to LVEF. The effect of a model-guided risk stratification strategy on clinical outcome should be investigated in a prospective trial.

## Disclosures

The authors have no conflicts of interest to disclose.
