# Supplementary material for: Predicting postinfarct ventricular tachycardia by integrating cardiac MRI and advanced computational reentrant pathway analysis
Source: Heart Rhythm. 2024 Oct;21(10):1962–9. doi: 10.1016/j.hrthm.2024.04.077 (PMC11739773; doi:10.1016/j.hrthm.2024.04.077)
Supplement: Supplemental Material [file mmc1.docx]

**Supplemental material**

**- Studied measures.** A comprehensive description of the image- and modelling derived parameters.

**- Table S1.** Comparison of imaging and simulation metrics between therapy vs. no-therapy groups, differentiated by primary or secondary prevention indication for ICD.

**Studied measures**

*Imaging*

1. The following measures have been derived semi-automatically using ADAS3D.
   1. Borderzone refers to the transition between healthy myocardium and scar core. This region exhibits a combination of structural and electrical remodeling and is characterized by altered electrophysiological properties compared to both healthy tissue and scar core. As previously described, borderzone plays a critical role in arrhythmogenesis and serves as a substrate for the initiation and maintenance of ventricular arrhythmias.
   2. Scar core refers to areas of irreversible myocardial damage, often characterized by fibrosis. These regions are completely devoid of functional myocardium and exhibit absence of electrical conduction properties. In the context of arrhythmias, scar core serves as substrate for reentrant circuits, contributing to the perpetuation of abnormal electrical activity.
   3. Imaging derived conduction corridors represent anatomical pathways, often borderzone surrounded by scar core, contributing to the formation of reentrant circuits and ventricular arrhythmias. The number and weight of conduction corridors indicates the quantity of these pathways and reflects their relative significance in generation and propagation of VT.
2. The following measures have been derived by analyzing the patient-specific enhancement model (generated in step 1) using an open-source meshing programme (Meshtool) and custom-written python scripts to create volumetric meshes containing separate tags for healthy, borderzone and scar core. Custom-written scripts were subsequently used to calculate the surface volume between these 3 tags to derive:
   1. the interface area between healthy myocardium and total enhancement, encompassing the volume of the boundary region where normal cardiac tissue transitions into areas of enhanced signal intensity, including both scar core and borderzone. This interface area represents the zone of interaction between structurally intact myocardium and regions of pathological remodeling or fibrosis, providing insights into the spatial distribution and extent of myocardial injury.
   2. the interface area between borderzone and core, referring to the volume separating the transitional zone of borderzone tissue from the central regions of scar core within the myocardium. This interface area delineates the interface between regions of partially preserved myocardial architecture and areas of complete myocardial replacement with fibrotic tissue.

*VITA*

1. Total number of VTs induced from all pacing sites

The total number of induced VTs refers to the cumulative count of distinct VT episodes that were initiated in VITA during the sequential pacing protocol from 17 sites (1 per AHA segment). The wavefront is examined to identify sites where the wavefront splits. These sites are considered electrically-isolated isthmuses through the scar. Subsequently, using VITA, uni-directional block is simulated from the isthmus exit/entrance site and the wavefront is restarted to derive the entire reentrant pathway involving the identified isthmus.

This step could be compared to the induction of VTs during electrophysiological studies and the resulting metric provides insight into the global arrhythmogenicity of the underlying substrate.

1. Number of unique VTs

Duplicate VTs, which utilize the same arrhythmic circuit, can be induced from different pacing sites but essentially represent the same underlying arrhythmia. Utilizing cyclically aligned activation time maps, duplicate VTs are filtered out to ensure that each unique VT is counted only once in the analysis.

1. Mean and maximum RTT

The round-trip-time (RTT) can be computed for each identified circuit and is the equivalent of VT cycle length. Mean RTT refers to the average duration of VT episodes across all identified circuits. On the other hand, maximum RTT denotes the longest duration of VT episodes observed among all identified circuits.

Therefore, mean RTT represents the average ‘cycle-length’ of VT episodes, while maximum RTT indicates the ‘longest cycle-length’ observed for the patient-specific computational model.

**Table S1.** Comparison of imaging and simulation metrics between therapy vs. no-therapy groups, differentiated by primary or secondary prevention indication for ICD.

| **ICD indication** | **Metrics** | | **Event** | **Event-free** |  |
| --- | --- | --- | --- | --- | --- |
| **Primary prevention** | **Imaging** | Borderzone (g) | 10.31 ± 3.8 | 9.46 ± 3.6 | p = .11 |
|  |  | Core (g) | 5.98 ± 3.7 | 5.73 ± 4.0 | p = .42 |
|  |  | Total corridors | 5.88 ± 7.2 | 4.82 ± 4.5 | p = .46 |
|  |  | Corridor weight (g) | 2.1 ± 1.7 | 1.62 ± 1.3 | p = .09 |
|  |  | Total enhancement (g) | 16.29 ± 6.6 | 15.08 ± 6.2 | p = .21 |
|  |  | Interface healthy-scar (cm^2^) | 83.14 ± 32.4 | 73.07 ± 24.5 | **p = .07** |
|  |  | Interface BZ-Core (cm^2^) | 58.88 ± 25.4 | 52.16 ± 21.8 | **p = .09** |
|  | **Simulation** | Total VTs | 39.31 ± 42.8 | 24.82 ± 24.4 | **p = .01*** |
|  |  | Unique VTs | 5.23 ± 4.5 | 3.71 ± 3.5 | **p = .02*** |
|  |  | Mean RTT (ms) | 84.15 ± 38.1 | 74.76 ± 41.6 | p = .12 |
|  |  | Maximum RTT (ms) | 124.96 ± 72 | 98.29 ± 61.2 | **p = .02*** |
| **Secondary prevention** | **Imaging** | Borderzone (g) | 11.53 ± 6.8 | 9.76 ± 4.3 | p = .85 |
|  |  | Core (g) | 8.39 ± 5.3 | 6.35 ± 3.9 | p = .28 |
|  |  | Total corridors | 4.5 ± 2.5 | 3.83 ± 1.8 | p = .64 |
|  |  | Corridor weight (g) | 1.99 ± 2.0 | 1.42 ± 1.5 | p = .28 |
|  |  | Total enhancement (g) | 19.92 ± 11.3 | 16.11 ± 7.3 | p = .49 |
|  |  | Interface healthy-scar (cm^2^) | 98.81 ± 39.7 | 89.82 ± 47.6 | p = .85 |
|  |  | Interface BZ-Core (cm^2^) | 73.53 ± 32 | 63.7 ± 36.7 | p = .66 |
|  | **Simulation** | Total VTs | 38.5 ± 30 | 43.67 ± 35.7 | p = .56 |
|  |  | Unique VTs | 5.14 ± 2.5 | 5.33 ± 2.5 | p = .95 |
|  |  | Mean RTT (ms) | 114.07 ± 55.8 | 89.67 ± 24.7 | p = .33 |
|  |  | Maximum RTT (ms) | 186.86 ± 120.2 | 148.33 ± 94.1 | p = .61 |

* denotes a significant difference (p < .05). BZ indicates borderzone; VT, ventricular tachycardia; RTT, round-trip-time.
